# Editorial: Dynamic Personality Science. Integrating between-Person Stability and within-Person Change

**DOI:** 10.3389/fpsyg.2017.01486

**Published:** 2017-09-08

**Authors:** Nadin Beckmann, Robert E. Wood

**Affiliations:** ^1^School of Education, Durham University Durham, United Kingdom; ^2^Australian Graduate School of Management, University of New South Wales Sydney, NSW, Australia

**Keywords:** personality, within-person, between-person, integrated approach, traits

“Personality is the dynamic organization within the individual of those psychophysical systems that determine his characteristic behavior and thought” (Allport, 1961, p. 28).

Trait theorists and social-cognitive theorists have begun to integrate their respective descriptions and explanations of personality. The new framing of personality accommodates both between-person stability and within-person variability in personality. Whilst individuals differ from each other in predictable ways—differences that can sufficiently be described by broad trait constructs such as neuroticism, conscientiousness, agreeableness, openness, extraversion, and core self-evaluations—they also vary systematically in the ways they respond to situations they encounter and change as a person over time. An integrated framework of personality raises many interesting questions. This Research Topic aims to move forward frontiers, both conceptually and empirically, for several of those questions. We provide new evidence in support of an integrated approach to personality, highlight currently active areas of research, and propose new directions of research into why individuals think, feel, and behave the way they do.

Research on the integrated approach to personality is now being conducted by research teams in Europe, the USA, and Australasia, much of which is captured by the papers in this Research Topic. Currently, there are several well-developed theoretical frameworks of integrated personality (e.g., Mischel and Shoda, 1995; Cervone, 2004; Fleeson and Jayawickreme, 2015) but empirical research is still in relatively nascent stages. There is a long way to go before the accumulated body of findings provide the level of robust support evident in trait research, particularly for the Big 5. Hopefully, the papers in this Research Topic, along with other similar offerings (e.g., special issues in European Journal of Personality, 2015; Journal of Research in Personality, 2015, Journal of Research in Personality, in press; Personality and Individual Differences, under review; Journal of Organizational Behavior, under review), will enable researchers to take stock and focus their research on the more important unanswered questions and speed up the accumulation of knowledge across different research agendas.

Initial steps toward the integration of traits and the systematic motivational dynamics of behavior tended to focus on the explanatory mechanisms for the impacts of traits on behavior using multi-trial experiments and field studies to capture the translation of traits into motivational states and responses. This research has a relative long tradition (e.g., Wood and Bandura, 1989) and is captured by two of the papers in this Research Topic (Cuadrado et al.; Hofmans et al.). More recently, the numbers of trials or measurement moments for within-person states have been extended through the use of digital technologies, which has enabled individual level modeling of the within-person dynamics. Several papers in this issue provide applications of individual level modeling in different areas of application, including the relational self (Andersen et al.), aesthetic appreciation (Fayn et al.), psychopathology (Wright et al.), situation change networks (Rauthmann and Sherman) and implicit theories (Cripps et al.).

As reflected in the opening quote, the papers in this Research Topic build on a tradition of studying personality from a within-person perspective that dates back at least to Allport (1937), who described a person-centered approach that focused on the organization of personality attributes within the individual and the development of the personality system over time (Allport, 1961). Allport's work was preceded by that of German psychologist Stern (1911), whose framework included *psychography*, which he described as the study of attributes within an individual (Asendorpf, 2015). Block (1971; Block and Block, 1980) drew a distinction between person-centered approaches that focus on the organization of traits or prototypes and variable-centered approaches that focus on the covariation of traits in the population. He identified personality prototypes derived from his theory of ego resiliency and ego control that described different configurations of Big 5 traits. The three personality prototypes identified by Block (1971), resilient, undercontrolled, and overcontrolled (also known as ARC types^1^), have been widely researched and are generally considered as having the strongest theoretical foundation and empirical rigor of existing typologies for the classification of individuals as personality types (Chapman and Goldberg, 2011).

The focus in this Research Topic is data *collected across occasions* at the within-person or intra-individual level (idiographic) to come to conclusions about groups of people or the population (nomothetic). The ARC types discussed by Asendorpf (2015) and others (e.g., Chapman and Goldberg, 2011) are not based on repeated observations of individuals. They base their conclusions on the same data as between-person trait analysis (in Stern's framework “correlation research”) but the data is analyzed differently using Q-sort or inverted factor analysis (in Stern's framework “comparative research”).

The new approaches to measurement of personality states have also led to the introduction of new statistical methods. The earlier approaches to the integration of stable between-person traits and dynamic within-person states relied on group-level methods such as repeated measures ANOVAs and SEM (e.g., Wood and Bandura, 1989; Cuadrado et al.), which are limited in their applicability to inferences about individuals. More recently, integrative approaches have employed growth curve modeling and Bayesian techniques (e.g., Cripps et al.; Hofmans et al.) to model repeated personality responses at the individual level.

These developments within personality psychology have run in parallel with new methodologies for studying the development of types, such as the ARC types (resilient, undercontrolled, and overcontrolled), in developmental psychology (Asendorpf, 2015), and personality assessment procedures in clinical psychology (Shedler and Westen, 2007). Asendorpf (2015) provides a critical review and recommendations for the methods and measures used in studies of ARC types that include assessments of the elevation, shape, and scatter of intra-individual personal profiles or configurations of traits. These include the Q-factor analyses pioneered by Block (1971), cluster analysis and more advanced methods such as latent cluster analysis (LCA). In clinical psychology, the Shefler-Westen Assessment Procedure (SWAP) provides clinicians with a diagnostic tool that integrates the scientific rigor of empirical approaches with the complexity and relevance required in clinical assessments. The SWAP includes a dictionary of 200 statements that provide detailed descriptions of diagnostic behaviors, including motives, functions, and contextual details, which enables clinicians to develop a profile of the patient using the Q-sort method. The SWAP has been shown to have good reliability, validity, and clinical utility (Shedler and Westen, 2007; Blagov et al., 2012) and is a measurement method that could be used in other applied areas, such as organizational psychology. However, as noted above, these data collection methods and analyses do not capture the intra-individual dynamics provided by data collected across occasions.

The areas of research identified by the papers in this Research Topic and requiring further attention include: The requirements for integration of between- and within-person factors; the conceptualization and operationalization of within-person units of personality; the study of within-person processes as antecedents and/or consequences of between-person individual differences; the comparison of within- vs. between-person personality structures and processes; the conceptualization, categorization, and measurement of situations; personality interventions based on the integrated approach; and data collection methods.

## Approaches to between- and within-person integration

A central objective of this Research Topic is to integrate and, where possible, to synthesize different conceptual and methodological approaches to the study of personality and their empirical outcomes. Work on the integrative perspective has been developing on several fronts, including: (1) A general acknowledgement that there is both stability and variability in personality and that it is worth studying both short- (state) and long-term (trait) personality change (e.g., Liu and Huang); (2) Comparison and the linking of findings from within- and between-person analyses (e.g., Wright et al.; Fayn et al.); (3) The conceptualisation of units of personality that are based on within-person data and represent individual differences in within-person structures and processes (e.g., Minbashian et al., 2010, in press); (4) Going beyond the descriptions of groups and individuals solely in nomothetic and idiographic terms, respectively (e.g., Lakey; Wright et al.; Cripps et al.).

Within-person refers to the analysis of structure and processes based on the repeated measurement of the same individual(s) over time and situations. The resulting data can be used to describe processes and structures that apply to a group of individuals (nomothetic, e.g., Minbashian et al., 2010) as well as single individuals (idiographic, e.g., Cripps et al.). Lakey demonstrates the integrated approach by using a variance partitioning approach to distinguish between Person effects (P), Situation effects (S), and P × S effects (i.e., individual profiles of responses across situations). Both S and P × S effects represent within-person variance (see also Wright et al. for nomothetic and idiographic within-person structures). Similarly, the process of transference outlined by Andersen et al. is thought to be common to all individuals (nomothetic within), whilst the underlying interpersonal knowledge structures are unique to single individuals (idiographic within). Cripps et al. demonstrate how individual growth curves (idiographic within) belong to groups based on implicit theories of ability (nomothetic).

More research on the nomothetic within-person effects that explain behavior is one path for gaining insights into general principles of personality that apply to individuals. This proposal is far from being new (e.g., see already Lamiell, 1981, 2013, 2014). However, there is now an increasing awareness of the necessity to study individuals repeatedly over time in order to adequately describe, explain, and predict the psychological processes underlying behavior (e.g., Roe, 2008, 2014; Grice, 2015; Grice et al., in press). This, together with the availability of new technology (e.g., apps and mobile devices) and statistical advances that enable researchers to collect and model extensive repeated measurement data more efficiently and effectively will allow researchers to make greater progress.

## Within-person units of personality

Investigation of the systematic components of within-person processes has included studies of stable between-person differences in within-person effects. Cripps et al. for example, identify different functional forms of the repeated responses for individuals with entity and incremental implicit theories (Dweck, 1999) and model their differential responses to performance setbacks. Contingent units of personality are another example. These studies are promising in that they provide evidence in support of the conceptualisation and operationalization of personality in terms of contingent, “if this …then that …, units” or behavioral signatures of the CAPS model (see Mischel and Shoda, 1995). Contingent units of personality are trait-like in that they are relatively stable between-person constructs, however, and in contrast to other traits such as the Big Five, they represent within-person structures and processes. Specifically, contingent units of personality describe (a) within-person variation in personality states as a function of within-person variation in situation perceptions, and (b) between-person differences in the strength and direction of within-person situation-state relationships. For instance, task-contingent conscientiousness refers to individual differences in the level with which one responds to increases in task demands with increases in state conscientiousness (Minbashian et al., 2010). In contrast to the conscientiousness trait, the focus is on a person's responsiveness to situational demands rather than their overall level of conscientiousness. Others have identified similar situation-response contingencies for other traits (e.g., Fleeson, 2007; Berenson et al., 2011; Huang and Ryan, 2011; Judge et al., 2014; Sherman et al., 2015). However, they still await wider replication. Little is known about their positioning within a nomological network and their predictive validity. To our knowledge only one study has shown that a contingent personality unit is correlated with a performance outcome variable (Minbashian et al., 2010).

Hofmans et al. provide novel findings into the functional forms of the contingent relationships modeled in the “if this …then that” units, which highlight the need for further research. Previous research has modeled the relationships in contingent units of personality as linear in form. Hofman and colleagues show that the relationship between work pressures and core self-evaluation (CSE) states is curvilinear. Hofman and colleagues' argument for an inverted U shaped functional form was based on an established body of evidence, that of how the impact of work pressure on performance follows an inverted U form (Yerkes and Dodson, 1908; Gardner and Cummings, 1988). Future research utilizing contingent units of personality will need to consider theoretical arguments and empirical evidence for different functional forms based on existing research evidence for the impacts of situational variables.

## Within-person processes

Within-person mechanisms underlying between-person differences, i.e., traits, have been studied extensively; and models of the within-person relationship between personality variables and relevant outcome variables, such as performance, have been devised for several traits. Many studies prioritize between-person differences by starting with well-established trait variables (e.g., Big Five) to then investigate underlying within-person processes that might explain why a specific trait is predictive of certain behavior. For example, Fayn et al. show that the personality domain Openness/Intellect reflects individual differences in aesthetic appreciation due to underlying appraisal-emotion contingencies that unfold at the level of the individual.

Studies have also uncovered the within-person mechanisms that link stable individual differences with behavior for traits outside the Big Five, including, in this Research Topic, dispositional prosocialness (Cuadrado et al.) and core self-evaluations (CSE, Hofmans et al.). Cuadrado et al. show how dipositional prosocialness and other dispositions are manifest as prosocial motivational states that predict levels of prosocial behavior. Consistent with theories of prosocial behavior and the conceptualizations of traits as individual differences in the sensitivity to situations (Marshall and Brown, 2006), Hofmans et al. and Cuadrado et al. provide some support for the moderation of relationships between traits and related reactions and responses. Hofmans and colleagues demonstrate that the sensitivity of individual CSE states to work pressure is moderated by levels of trait CSE. For those with low trait CSE “the depleting effect of work pressure via state CSE happens at low levels of work pressure, while for people high in trait CSE the depleting effect is located at high levels of work pressure” (Hofmans et al.).

Other authors prioritize within-person processes and start with process-level variables as antecedents of between-person individual differences. The work by Andersen et al. on the relational self provides a good example of such an approach in their application of the CAPS model of Mischel and Shoda (1995) to the interpersonal domain. The authors outline how idiosyncratic within-person knowledge structures and processes might explain the emergence of stable, between-person differences as described by specific traits such as rejection sensitivity, and potentially—though this is an open empirical question—more global interpersonal traits such as agreeableness and extraversion. Whilst it seems sensible to use the Big Five as an organizing framework from where to start (top-down), this might also be limiting. Starting with process-level investigations (bottom-up) might lead to the emergence of traits that are not covered by the Big Five. Thus, we suggest the top-down approach be complemented by a bottom-up approach.

## Comparisons of within-person and between-person personality structure and processes

Between-person findings are often used as proxies for within-person phenomena even though it has been repeatedly demonstrated that this is inappropriate (see Lamiell, 1981, 2014; Nezlek, 2001; Borsboom et al., 2003; Molenaar, 2004; Schmitz, 2006; Grice, 2015). Within-person structures and processes may not be the same as those identified for related traits (e.g., Borkenau and Ostendorf, 1998; Grice et al., 2006; Beckmann et al., 2010). Wright et al. investigate the structure of psychopathology in individuals with personality disorder. Whilst at the between-person level they found a two-dimensional structure comprising the widely accepted broad dimensions of mental disorder (internalizing, externalizing), findings at the within-person level suggested a more differentiated four-dimensional structure of psychopathology (negative affect, detachment, hostility, impulsivity). Building on recent developments in structural equation modeling (e.g., unified SEM) they also demonstrate how modeling of the dynamic patterns of daily responding for individuals provides information about the person's psychological functioning of relevance to clinicians (see also Rauthmann and Sherman, for individual-specific situation change networks).

Another frontier is the type of statistical methods used to model within-person processes. Two of the papers in this issue use Bayesian techniques to model within-person processes (Cripps et al.; Hofmans et al.). Bayesian techniques are not widely used or understood in psychology and are not yet available in easy to use packages but offer additional flexibility in the modeling of complex and dynamic response patterns at the level of the individual. For example, they can provide an estimate of the probability with which each individual in a sample conforms to the proposed hypothesis or model. Also, because they do not assume asymptotic normality of the sample estimates, inferences about patterns of individual responding can be made based on relatively few observations. As Bayesian and other statistical techniques become more widely available, there is no excuse for not collecting intensive repeated measurement data and modeling within-person processes longitudinally and at the level at which they occur—the individual.

## Situations

Situations are central to an integrated view on personality because of the impacts they have on within-person variations in cognitive, affective and behavioral responses. This has become a very active area of research (e.g., Wood et al., 2011; Rauthmann et al., 2015). The recently introduced taxonomies provide personality researchers with tools to conceptualize, categorize, and measure situations, and a language to communicate about situations, both with regard to objective features (PERLS, Noftle and Gust, 2015) and subjective perceptions of situations (DIAMONDS, Rauthmann et al., 2014; Rauthmann and Sherman, 2016; CAPTION, Parrigon et al., 2017). An alternative approach to the study of situations is to focus on specific domains (e.g., tasks or interpersonal domains), and study them at a more fine-grained (e.g., facet) level. In the task domain, for example, this may include perceived task support, task difficulty, task urgency, task importance, and task controllability (Minbashian et al., 2010; see Judge and Zapata, 2015, for work-related context variables, and Wood (2005, Table 1) for categories of organizational events and work arrangements that stimulate and facilitate self-regulatory processes).

Regardless of the degree of specificity, study of the dynamic components of personality requires an understanding of how and why situations and perceptions of situations change and how these changes relate to short- and long-term changes in personality. Rauthmann and Sherman outline a comprehensive research programme to study situation change, including person-situation transactions, and provide preliminary data illustrating their approach at both the between- and within-person level of analysis.

Clearly, the choice of situational variables will need to be based on the theoretical arguments that link situations to responses of interest. For example, perceived collaboration in a team setting could be expected to reduce the risks associated with extraverted responses such as gregariousness and contributions to conversations. Therefore, on average, this would produce a positive relationship between perceived collaboration and state extraversion. However, assessments of risk may differ between individuals and lead to different scores for the collaboration-extraversion relationship. Established group level predictors for response states of interest—such as perceived justice as a predictor of compliance with requests—will provide a useful source of ideas for the situational variables to study.

We now consider two areas of research that we think deserve more attention. These are the malleability of personality (including the question of how to bring about personality change), and the measurement of dynamic components of personality.

## Personality malleability and personality interventions

Topics might include the trainability of personality, personality change in response to life events (e.g., work, schooling), and personality interventions for clinical and non-clinical samples (e.g., in educational, organizational settings). Whilst there is now considerable evidence of short-term within-person variability in personality states (e.g., Fleeson, 2001; Fleeson and Gallagher, 2009; Judge et al., 2014; Fleeson and Jayawickreme, 2015; Fleeson and Law, 2015), a growing literature provides evidence about long-term within-person change in personality traits in response to changes in life circumstances (e.g., Lüdtke et al., 2011; Bleidorn et al., 2016; Niehoff et al., 2017; for short-term trait change see Shields et al., 2016). For example, Liu and Huang studied personality change in the context of cross-cultural adjustment. They show that both the initial level of contextualized extraversion as well as the rate of observed change in extraversion in response to new cultural experiences predicted adjustment outcomes. Evidently, personality change was an important precursor of transition success. Individual differences in personality malleability, i.e., flexibility in personality responding, might indicate an underlying ability that enables some individuals to better adapt and adjust to various changes in life circumstances than others. To date, there are few studies of how insights about malleability can be used for training and other interventions targeting personality change. Hudson and Fraley (2015), for example, show that people can actively change their personality traits, if they are motivated to do so, and such change can be facilitated by carefully designed interventions. Hermsen et al. (2016) give an example of how providing feedback through digital technology can be used to disrupt and change habits, which may also be employed in reshaping the contingent units of personality that predict targeted behaviors.

## Measurement considerations

Mill et al. provide a timely reminder of potential biases in the measurement of emotions, one of the core responses in units of personality. They show how ratings of emotions that require retrospective recall ranging from 1 day to 2 weeks are more negative for older people and for those who are more tired at the time of the data collection, when compared to those who are younger and less tired. They also found that recollections of fear, sadness, anger, and happiness emotions were related to selected Big Five personality traits. The Mill et al. findings highlight the need for measurement of both trait and state emotions to take account of a range of potential biases in responses. Experience sampling measures of emotions that ask for a daily recall may also differ in systematic ways from those that collect more immediate responses multiple times each day.

More generally, work is needed to establish the psychometric quality of experience sampling personality state measures. Researchers interested in the measurement of personality traits will find a number of validated instruments. These are much harder to find for the measurement of personality states using experience sampling designs in the field (see Finnigan and Vazire, 2017, for a recent validation study). An additional complication is that the number of items that can reasonably be presented to participants on a daily basis has to be relatively small, providing less room for exploration and testing of new questions. It also means that often only a subset of items is taken from established measures.

Second, and following from the first point, research should test the validity of more efficient measurement procedures for the collection of data used to assess dynamic components of personality. The experience sampling method that is commonly used to collect within-person data is a labor intensive procedure that might extend over several weeks. Participants sometimes find the daily requests for responses intrusive and they might not respond if they are engaged in an activity. Thus, it is worth testing alternative approaches and their validity. For example, the semantic sequential priming task might be a more efficient method to assess contingent units of personality (Moeller et al., 2010; Berenson et al., 2011).

These are exciting times for personality researchers. The integration of trait and social-cognitive theories promises to bring about a more differentiated understanding of personality, it also highlights where “blind spots” have been and data is still scarce or missing. There is also the added advantage of considering personality as a phenomenon that is, at least in principle, malleable. As more data become available on how to facilitate personality change, psychologists will be better able to support individuals to become the person they aspire to be. It is here where personality research might be most relevant and have lasting impact outside the ivory towers of academia.

In conclusion, the papers in the current issue highlight both progress toward and areas requiring further research for an integrated approach to personality. Hopefully, researchers across the different sub-disciplines of psychology, including health, educational, and organizational psychology will find ideas in the theories and approaches outlined in the papers in this Research Topic and beyond to inform their own research.

## Author contributions

All authors listed have made a substantial, direct and intellectual contribution to the work, and approved it for publication.

### Conflict of interest statement

The authors declare that the research was conducted in the absence of any commercial or financial relationships that could be construed as a potential conflict of interest.

## Footnotes

^1^ARC refers to Asendorpf-Robins-Caspi, who were the lead authors of three articles that reported the initial extensions of Block's types (Caspi and Silva, 1995; Robins et al., 1996; Asendorpf et al., 2001).
